# Gut Microbiota Dysbiosis in Patients with Biopsy-Proven Nonalcoholic Fatty Liver Disease: A Cross-Sectional Study in Taiwan

**DOI:** 10.3390/nu12030820

**Published:** 2020-03-19

**Authors:** Ming-Chao Tsai, Yu-Yin Liu, Chih-Che Lin, Chih-Chi Wang, Yi-Ju Wu, Chee-Chien Yong, Kuang-Den Chen, Seng-Kee Chuah, Chih-Chien Yao, Pao-Yuan Huang, Chien-Hung Chen, Tsung-Hui Hu, Chao-Long Chen

**Affiliations:** 1 Division of Hepato-Gastroenterology, Department of Internal Medicine, Kaohsiung Chang Gung Memorial Hospital and Chang Gung University College of Medicine, Kaohsiung, 123 Ta Pei Road, Niao Sung Dist., Kaohsiung City 83301, Taiwan; chuahsk@seed.net.tw (S.-K.C.); chihchienyao@gmail.com (C.-C.Y.); paoyuan813@gmail.com (P.-Y.H.); e580306@ms31.hinet.net (C.-H.C.);; 2 Graduate Institute of Clinical Medical Sciences, Chang Gung University College of Medicine, Kaohsiung 83301, Taiwan; 3 Department of General Surgery, Kaohsiung Chang Gung Memorial Hospital and Chang Gung University College of Medicine, Kaohsiung 83301, Taiwan; liuyuyin5750@gmail.com; 4 Liver Transplantation Center and Department of Surgery, Kaohsiung Chang Gung Memorial Hospital and Chang Gung University College of Medicine, Kaohsiung 83301, Taiwan; immunologylin@gmail.com (C.-C.L.); ufel4996@gmail.com (C.-C.W.); wuyiju0904@gmail.com (Y.-J.W.); clchen@cgmh.org.tw (C.-L.C.); 5 Center for Translational Research in Biomedical Sciences, Liver Transplantation Program and Department of Surgery, Kaohsiung Chang Gung Memorial Hospital and Chang Gung University College of Medicine, Kaohsiung 83301, Taiwan; dennis8857@gmail.com

**Keywords:** gut microbiota, nonalcoholic fatty liver disease (NAFLD), nonalcoholic fatty liver (NAFL), nonalcoholic steatohepatitis (NASH), *Ruminococcus*

## Abstract

The gut microbiota plays a role in nonalcoholic fatty liver disease (NAFLD), but data about gut dysbiosis in Asians with NAFLD remains scarce. We analyzed the differences in fecal microbiota between adults with and without NAFLD. This cross-sectional study examined adults with histology-proven NAFLD (25 nonalcoholic fatty liver (NAFL) patients, 25 nonalcoholic steatohepatitis (NASH) patients, and 25 living liver donors (healthy controls)). The taxonomic composition of the gut microbiota was determined by 16S ribosomal RNA gene sequencing of stool samples. The NAFL and NASH groups showed lower total bacterial diversity and richness than the controls. NAFLD patients had higher levels of the phylum Bacteroidetes and lower levels of Firmicutes than controls. The genus *Ruminococcaceae UCG-010*, family *Ruminococcaceae*, order *Clostridiales*, and class *Clostridia* were less abundant in patients with NAFL or NASH than healthy individuals. The lipopolysaccharide biosynthesis pathway was differentially enriched in the NASH group. This study examined the largest number of Asian patients with biopsy-proven NAFL and NASH in terms of dysbiosis of the gut microbiota in NAFLD patients. NAFLD patients had higher levels of Bacteroidetes and lower levels of Firmicutes. These results are different from research from western countries and could provide different targets for therapies by region.

## 1. Introduction

Nonalcoholic fatty liver disease (NAFLD) is defined by the presence of at least 5% hepatic steatosis with a lack of common causes of secondary hepatic fat accumulation, such as excessive alcohol consumption, chronic viral hepatitis, autoimmune hepatitis, or long-term use of steatosis-inducing medications. NAFLD is the most common form of chronic liver disease in many industrialized counties, and globally, almost one-quarter of adults show excessive hepatic fat accumulation [1,2]. The condition constitutes a spectrum ranging from hepatic steatosis (nonalcoholic fatty liver (NAFL)) to steatosis with hepatocellular injury and possibly fibrosis (non-alcoholic steatohepatitis (NASH)). NASH is the aggressive form of NAFLD. It leads to liver fibrosis and finally cirrhosis and hepatocellular carcinoma, which increase liver-related morbidity and mortality [3]. NAFLD is strongly associated with obesity, diabetes, and insulin resistance, along with other features of metabolic syndrome. For many decades, NAFLD was thought to be predominantly driven by obesity in the context of genetic susceptibility. However, this association with obesity does not explain the variable severity of the illness and its outcomes [4]. The pathogenesis of NAFLD is not entirely understood. It is thought to involve complex interactions, including genetic factors, multiple dietary factors, and environmental factors, and more recently, microbial attacks on the liver have been recognized [5,6].

Recently, the gut microbiota has gained great attention in metabolic disease since gut dysbiosis has been demonstrated in obesity [7,8], diabetes [9], metabolic syndrome [10,11], and cardiovascular disease [12]. Dysbiosis is defined as any change in the composition of the microbiota other than that commonly found in healthy conditions. The human gut is home to around 100 trillion commensal organisms that carry 150 times more genes than the host and are collectively referred to as the microbiome [9]. Animal experiments in which the microbiome has been manipulated have shown that the gut microbiota plays an important role in the pathogenesis of NAFLD [13,14]. Nevertheless, only a few studies have linked gut dysbiosis with the severity of NAFLD in humans [15,16,17,18]. These studies had small sample sizes, heterogeneous populations, and divergent tools for gut microbiota analysis. Furthermore, not all patients underwent a liver biopsy, which may have resulted in the under-diagnosis of NASH. All of these studies had limited assessments of the association between gut dysbiosis and NAFLD. 

Recently, Boursier et al. reported on the severity of NAFLD in association with gut dysbiosis in a correctly diagnosed population of biopsy-proven NAFLD patients [19]. NASH was independently and positively associated with higher levels of the genus *Bacteroides* in the fecal microbiome. However, all included patients were from a western country (Angers, France). The large-scale Human Microbiome Project (HMP) [20] reported that race was strongly correlated with specific microbial species and indicated that there are differences in microbiota composition between eastern and western countries. To date, there has been a marked paucity of studies on the role of gut microbiota in biopsy-proven NAFLD patients, especially in Asians. The aim of our study was to evaluate the changes in fecal microbiota in a population of biopsy-proven NAFL and NASH patients along with healthy controls from Taiwan.

## 2. Materials and Methods

### 2.1. Patients and Control Subjects 

This study was approved by the Institutional Review Broad of Chang Gung Memorial Hospital (No.201701911B0) and was conducted in accordance with the Declaration of Helsinki and current ethical guidelines. A total of 50 biopsy-proven NAFLD patients (25 with NAFL and 25 with NASH) and 25 biopsy-proven healthy subjects (non-NAFLD) were recruited. After providing adequate explanation, written informed consent for participation in this study was obtained from all the subjects prior to the sample collection. The participants were asked to provide a stool sample on the morning of their liver biopsy, while a blood sample was checked to determine metabolic and hepatic parameters. Healthy subjects who were assessed for liver donation at Kaohsiung Chang Gung Memorial Hospital were screened as controls. 

The inclusion criteria were subjects aged 18–70 years old with histology-proven NAFLD or healthy liver. One of the exclusion criteria was a history of another hepatic disease, such as chronic hepatitis B, hepatitis C, autoimmune hepatitis, primary biliary cirrhosis, sclerosing cholangitis, hemochromatosis, or Wilson’s disease, or excessive alcohol consumption (>140 g/week in men or >70 g/week in women). The other exclusion criteria were liver decompensation or malignancy, a history of chronic inflammatory bowel disease or bariatric surgery, and treatment with antibiotics or proton pump inhibitors within three months before inclusion. 

### 2.2. Liver Histology

All biopsies were evaluated by two experienced hepatopathologists who were blinded to the clinical characteristics. NASH was diagnosed according to Brunt et al. [21] and required the presence of steatosis, ballooning, and lobular inflammation. The NAFLD activity score (NAS) was assessed for each biopsy specimen according to the guidelines established by Kleiner et al. [22] Steatosis was graded with 0–3 points as follows: (0) steatosis <5%; (1) 5–33%; (2) steatosis 33–66%; and (3) steatosis >66%. Lobular inflammation was scored as follows according to the number of foci of inflammatory infiltration per 200× microscopic field: (0) no inflammation; (1) <2 foci; (2) 2–4 foci; and (3) >4 foci. Ballooning degeneration was scored between 0 and 2 points as follows: (0) none; (1) few balloon cells; and (2) many balloon cells. NAS was defined as the sum of the scores ranging from 0 to 8 and is proposed to be a measure of overall activity of the disease. 

### 2.3. Stool Sample Preparation and 16S rRNA Gene Amplification and Sequencing 

Fresh stool samples were collected at home from study population using separate sterile feces containers containing stabilizer (DNA/RNA Shield^TM^, ZYMO RESEARCH CORP, Irvine, CA, USA). Samples with liquid stabilizer were shaken vigorously immediately after collection and stored in ambient temperature. The samples were transferred to the laboratory of Biotools Co., Ltd. (Taipei, Taiwan) within one week and then all samples were stored at −80 °C. Total genomic DNA from samples was extracted using the column-based method (e.g., QIAamp PowerFecal DNA Kit, Qiagen, Austin, TX, USA). DNA concentration was determined and adjusted to 5 ng/µL for the following process. For the 16S rRNA gene sequencing, V3–-V4 region was amplified by specific primer set (319F: 5′-CCTACGGGNGGCWGCAG-3′, 806R: 5′- GACTACHVGGGTAT CTAATCC-3′) according to the 16S Metagenomic Sequencing Library Preparation procedure (Illumina). In brief, 12.5 ng of gDNA was used for the PCR reaction carried out with KAPA HiFi HotStart ReadyMix (Roche, Cape Town, South Africa) under the PCR condition: 95 °C for 3 min; 25 cycles of: 95 °C for 30 s, 55 °C for 30 s, and 72 °C for 30 s; 72 °C for 5 min and hold at 4 °C. The PCR products were monitored on 1.5% agarose gel. Samples with bright main strip around 500 bp were chosen and purified by using the AMPure XP (Beckman, Indianapolis, IN, USA) beads for the following library preparation. The Sequencing library was prepared according to the 16S Metagenomic Sequencing Library Preparation procedure (Illumina). In brief, a secondary PCR was performed by using the 16S rRNA V3–V4 region PCR amplicon and Nextera XT Index Kit (Illumina, San Diego, CA, USA) with dual indices and Illumina sequencing adapters (Illumina). The indexed PCR product quality was assessed on the Qubit 4.0 Fluorometer (Thermo Scientific, Waltham, MA, USA) and Qsep100^TM^ system (BIOptic, Taipei, Taiwan). Equal amounts of the indexed PCR product were mixed to generate the sequencing library. Finally, the library was sequenced on an Illumina MiSeq platform (Illumina, San Diego, CA, USA) and paired 300-bp reads were generated.

### 2.4. Processing and Analysis of Gut Microbiota

Quality filtering of the raw tags was performed under specific filtering conditions according to Quantitative Insights Into Microbial Ecology (QIIME) version 2 [23]. Sequences with 97% resemblance or greater were categorized as having the same operational taxonomic units (OTUs) using the USEARCH algorithm. The relative abundance of each taxon was determined by dividing the assigned read counts by the total read counts. The alpha (Chao 1 and ACE) and beta (PLS-DA) diversity of the gut microbiota was calculated using the relative abundance of OTUs in each sample and QIIME (Version 2). A heat map of the identified key variables was completed using Heat Map Builder (pheatap, https://CRAN.R-project.org/package=pheatmap).

The linear discriminant analysis (LDA) effect size (LEfSe) method was used to determine biomarkers to identify the differential enrichment of abundant taxa between groups. The algorithm performs a nonparametric factorial Kruskal–Wallis sum-rank test and LDA to determine statistically significant different features among taxa and estimates the effect size of the difference [24]. Differences were considered significant with adjusted *p* values <0.05, and logarithmic LDA scores greater than 4 were displayed in the final output. 

The functional composition of metagenomes was predicted from 16S rRNA data by Phylogenetic Reconstruction of Unobserved States (PICRUSt) software, as described previously [25]. The Kyoto Encyclopedia of Genes and Genomes (KEGG) ortholog abundances were used to design the map of metabolic pathways (KEGG database, http://www.genome.jp/kegg/).

### 2.5. Statistical Analysis 

Baseline characteristics and clinical variables were summarized as the mean ± standard deviation, the median (25th–75th percentile), or a percentage. Original observation data in taxa summary plots were normalized by computing the relative abundance. The chi-squared test, an independent two-sample *t*-test, and Kruskal–Wallis test were used as appropriate to assess the significance of any differences in distributions. Statistical significance was accepted with a two-sided *p* value of <0.05. Statistical analyses were performed using SPSS version 18.0 for Windows (SPSS Inc., Chicago, IL, USA).

## 3. Results

### 3.1. Clinical Characteristics of Healthy Individuals and Patients with NAFLD

A total of 75 subjects were enrolled in this study, including 25 health controls (non-NAFLD group), as well as 25 NAFL patients and 25 NASH patients (NAFLD group). The characteristics and clinical data are shown in Table 1. The patients with NAFLD (NAFL or NASH) were older than the healthy controls. There was no significant difference in the gender among groups.

As expected, NAFLD patients had significantly higher metabolic parameters than the healthy controls, including body mass index (BMI), fasting glucose, glycohemoglobin, total cholesterol, total triglycerides, and homeostatic model assessment of insulin resistance (HOMA-IR). In the histology analysis, NAFLD patients had significantly higher rates of steatosis, fibrosis stages, and NAS. Among patients with NAFLD, NASH patients had significantly higher triglyceride levels, fasting glucose levels, HbA1C levels, and HOMA-IR than those with NAFL. As expected, NASH patients had higher rates of steatosis and NAS and higher fibrosis stages than those with NAFL.

### 3.2. Gut Microbiota in NAFL/NASH and Healthy Controls

We characterized the fecal microbiomes of a total of 75 samples. After applying strict trimming criteria to exclude low-quality reads, a total of 5,083,499 high-quality sequences were obtained with an average of 67,780 reads per sample (range: 33,723–139,557 reads per sample). The rarefaction curve analysis suggested that the microbiome diversity was captured beyond 30,000 reads across the whole sample as the number of observed species plateaued (Figure S1A). 

**Figure 1:**
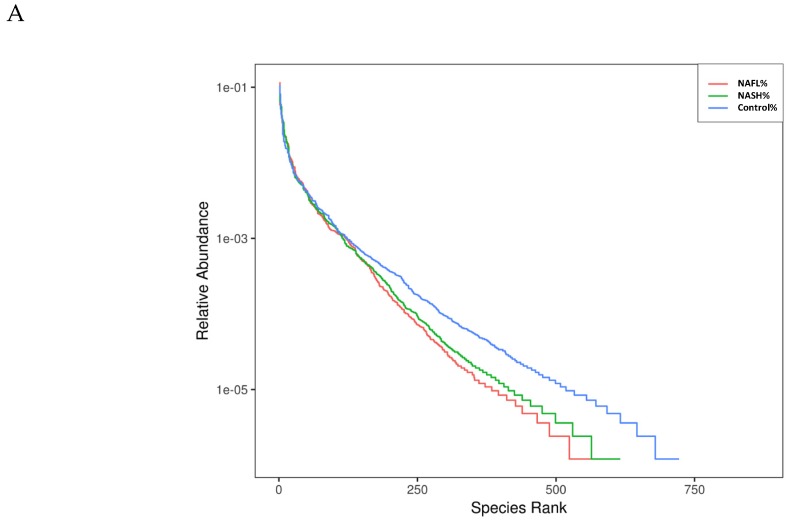

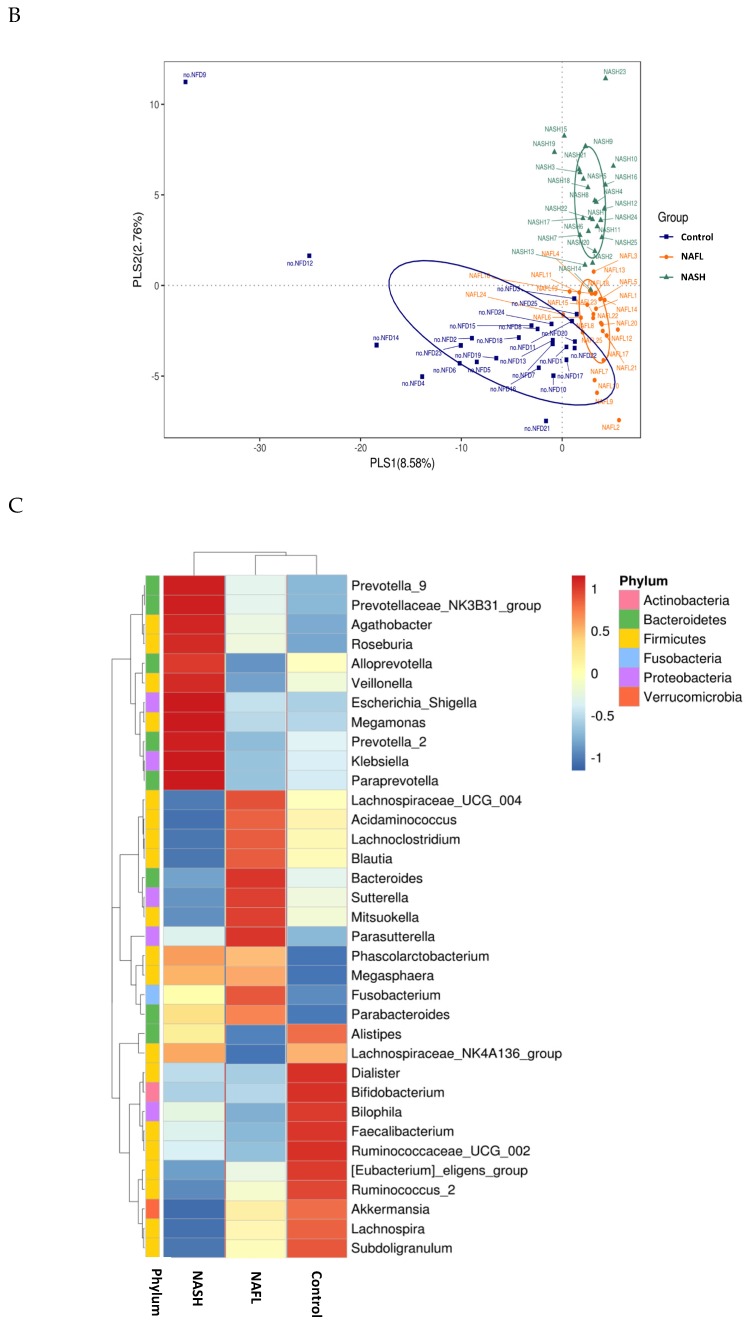
The diversity in gut microbiota of healthy individuals and patients with nonalcoholic fatty liver disease (NAFL) or nonalcoholic steatohepatitis (NASH). (**A**) Observed species numbers in the three groups. (**B**) β-diversity changes in gut microbiota across groups by the partial least squares discriminant analysis (PLS-DA), which presented changes between samples collected from the control (non-NAFLD), NAFL, and NASH groups. Each node represents each sample. Control (non-NAFLD), NAFL, and NASH subjects are colored in blue, orange, and green, respectively. (**C**) Heat map of the top 35 genera among groups.

The overlapping OTUs of the three groups is shown in a Venn diagram in Figure S1B. The data demonstrate that the healthy controls, NAFL patients, and NASH patients independently had 130, 40, and 33 OTUs, respectively. According to the rank-abundance curves, the species richness of the healthy controls was higher than that of the NAFL and NASH groups (Figure 1A). The indices of alpha-diversity, including Chao1 and ACE, were not significantly different among the three groups. We also evaluated the compositions of the gut microbiota among the three groups using the score plots of the partial least squares discriminant analysis (PLS-DA) method (Figure 1B). The plots showed that there was a significant separation between each group with 8.58% and 2.76% explanation of the variation of the gut microbiota composition in PLS1 and PLS2, respectively. Figure 1C shows a heat map of the top 35 genera, which reveals the very diverse patterns among the three groups. To summarize, the findings indicate distinct microbes among the three groups. Briefly, there is dysbiosis in the composition of the gut microbiota among NAFL patients, NASH patients, and healthy individuals.

### 3.3. Bacterial Abundance and Distribution of the Predominant Bacteria at Different Taxonomic Levels

**Figure 2:**
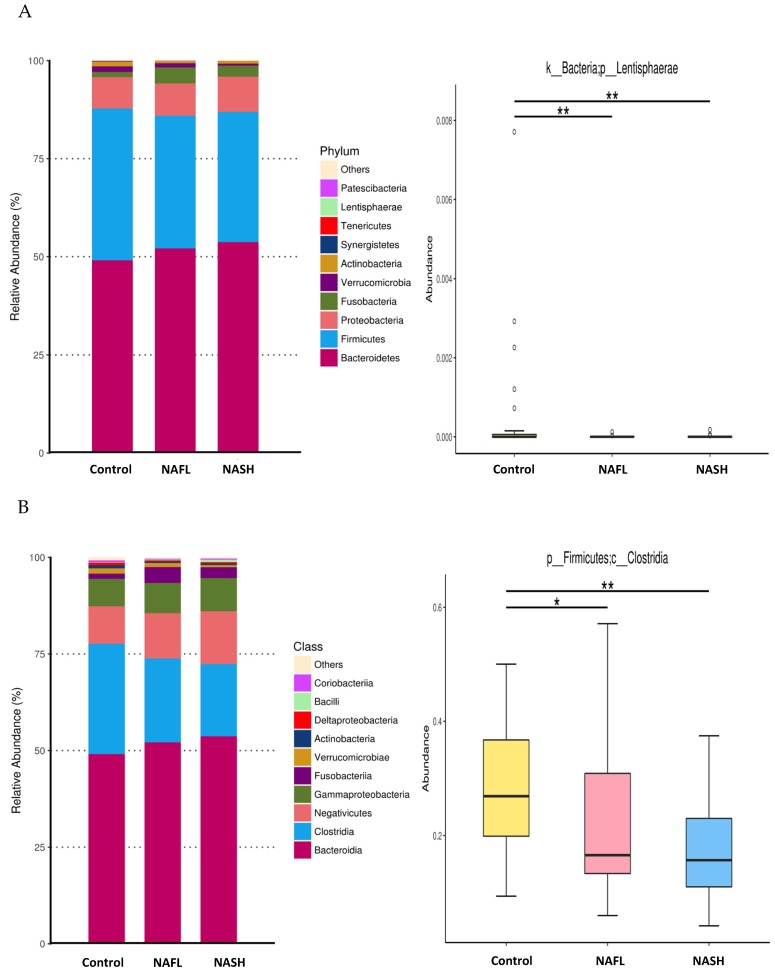

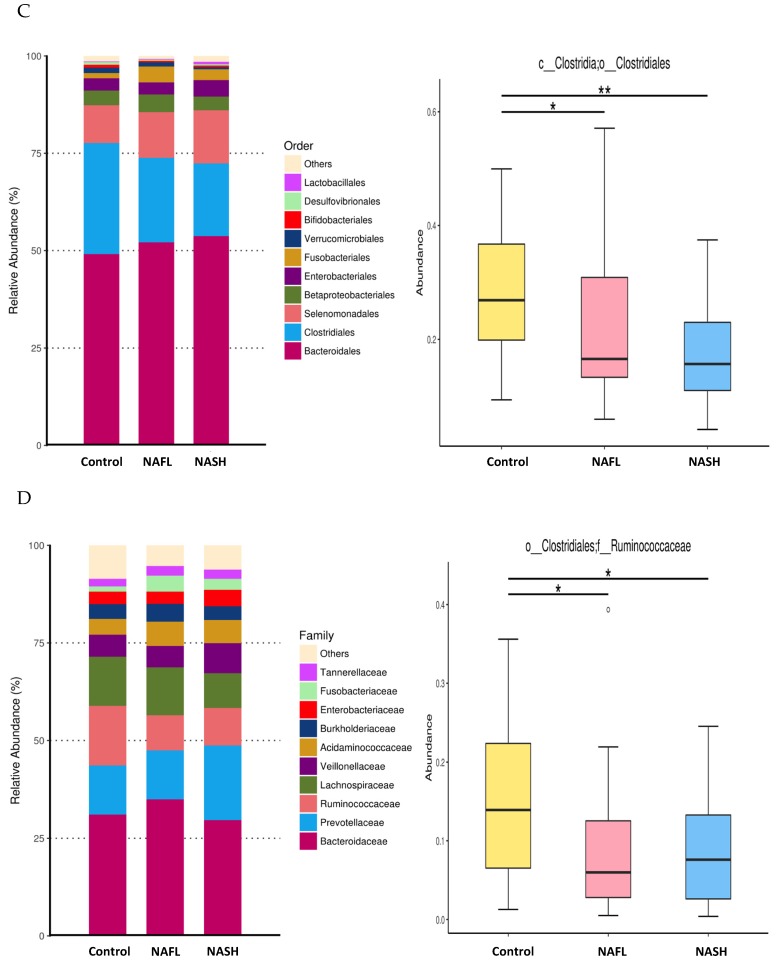

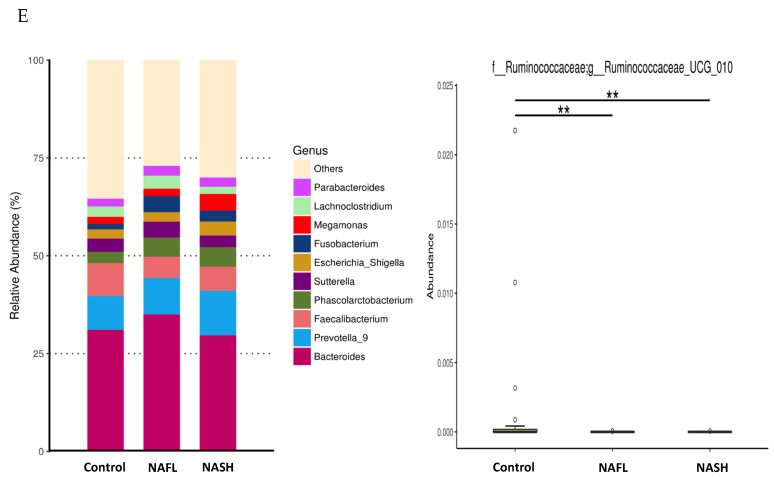
Relative abundance of the top 10 microbiota and significantly scarce bacteria in the NAFL, and NASH groups compared with the healthy controls at the phylum (**A**), class (**B**), order (**C**), family (**D**), and genus (**E**) levels. Boxplots shows the median, the 25th, and the 75th percentile of relative abundances of bacteria. *p < 0.05 and ** *p* < 0.01 vs. healthy controls, Kruskal–Wallis test.

In the gut microbiomes examined, 16 bacteria phyla, 24 classes, 41 orders, 70 families, and 228 genera were identified. Figure 2 shows the bacterial distribution, which is characterized by the relative abundances of the top 10 of each taxon. At the phylum level (Figure 2A and Table S1), the main phyla were Bacteroidetes and Firmicutes, together accounting for nearly 90% of the total sequences. Of these, the abundance of Lentisphaerae was significantly lower in the NAFL and NASH groups than in the healthy group (*p* < 0.01). The ratio of Firmicutes to Bacteroidetes had been reported as a biomarker for obesity, but there was no significant difference between each group (Figure S2). 

At the class level (Figure 2B and Table S2), the most prevalent classes were Bacteroidia, Clostridia, Negativicutes, and Gammaproteobacteria. Of these, Clostridia was significantly less abundant in the NAFL (*p* < 0.05) and NASH groups (*p* < 0.01) than the healthy controls. At the order level (Figure 2C and Table S3), the most prevalent were Bacteroidales, Clostridiales, Selenomonadales, Betaproteobacteriales, and Enterobacteriales. Of these, Cloastridiales was significantly less abundant in the NAFL (*p* < 0.05) and NASH groups (*p* < 0.01) than the healthy group. At the family level (Figure 2D and Table S4), the most prevalent families were Bacteroidaceae, Prevotellaceae, Ruminococcaceae, Lachnospiraceae, Veillonellaceae, Acidaminococcaceae, Burkholderiaceae, and Enterobacteriaceae. Ruminococcaceae was significantly less abundant in the NAFL and NASH groups (*p* < 0.05) than the healthy group. At the genus level (Figure 2E and Table S5), the most abundant genera were *Bacteroides, Prevotella,* and *Faecalibacterium*. *Ruminococcaceae UCG-010* was significantly less abundant in the NAFL and NASH groups (*p* < 0.01) than the healthy controls. It should be noted that the genus Ruminococcaceae UCG-010 belongs to the family Ruminococcaceae, order Clostridiales, and class Clostridia, and all were less abundant in patients with NAFL or NASH than healthy controls.

### 3.4. Differential Microbiota Compositions among Healthy and NAFL/NASH Groups

**Figure 3:**
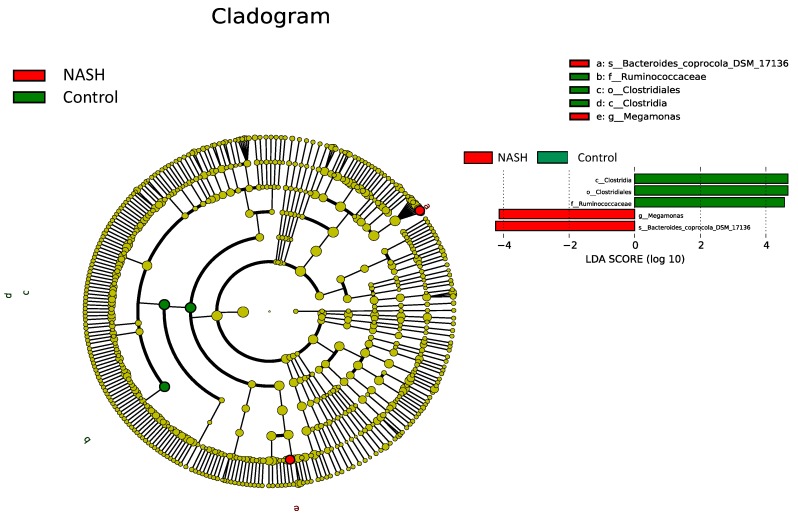
Linear discriminant analysis effect size (LEfSe) comparison of gut microbiota among healthy, NAFL, and NASH groups. The cladogram displays the taxonomic tree of differentially abundant taxa. The histogram represents the linear discriminant analysis (LDA) scores of bacteria with significant differential abundance between the compared groups identified by different colors.

LEfSe modeling with a logarithmic LDA score cutoff ≥4.0 was then performed to confirm both the statistical and taxonomic differences between the gut microbiota of patients with NAFL or NASH and that of healthy controls, as shown in Figure 3. The NASH patients had greater abundance of the genus *Megamonas* and species *Bacteroides coprocoia* DSM 17136, whereas greater abundance of the class Clostridia, order Clostridiales, and family Ruminococcaceae were found in healthy controls. These results are compatible with the previous abundance analysis at different levels (Figure 3).

### 3.5. Microbial Functional Dysbiosis in Metabolic Pathways in NAFLD

**Figure 4:**
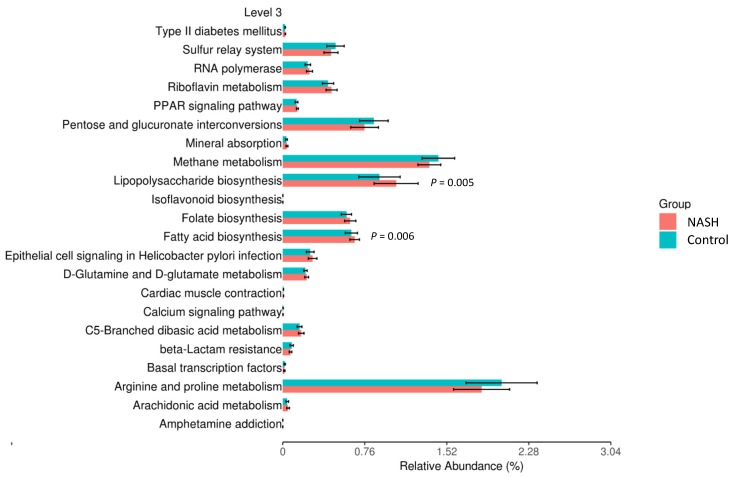
Difference in relative abundance of predicted microbial genes related to metabolism information between NASH and healthy controls. Data were processing by 16S rRNA sequencing data with PICRUSt using level 3 of Kyoto Encyclopedia of Genes and Genomes (KEGG) orthologs using Student’s *t*-test.

To further understand the metabolic functions of genera among NAFLD patients and healthy controls, we inferred the metagenomes from the 16S rRNA data using PICRUSt [26] using level 3 of the Kyoto Encyclopedia of Genes and Genomes database (KEGG) orthologs to gain insight into the functional and metabolic changes of the microbial communities between NAFLD patients and healthy controls. For the healthy and NASH groups (Figure 4), there are many significant metabolic pathways; notably, lipopolysaccharide (LPS) biosynthesis (*p* = 0.005) and fatty acid biosynthesis (*p* = 0.006) appeared to be over-represented in the microbiomes of NASH patients, which were mainly related to glycan biosynthesis and metabolism and lipid metabolism, respectively. Collectively, the present data suggest that the change of the bacterial composition can lead to significant changes in gene function, which might contribute to the development of NAFL or NASH by the metabolic pathways, although it remains unclear whether LPS is increased in NASH.

## 4. Discussion

NAFLD is a disease of increasing interest as its prevalence is on the rise, affecting a quarter of the global population. This makes it the most common chronic liver disease in developed countries [27]. Traditionally, NAFLD has been considered a problem in western countries, but it is now increasingly recognized in Asian populations. Recently, many studies have implicated the gut microbiota as a leading cause in the context of NAFLD and NASH [13]. Data from human studies have established a relationship between the gut microbiota and NAFLD, but there have been few studies on Asians, and they have yielded inconsistent results. In the present study, we have demonstrated the altered diversity and composition of the gut microbiota of patients with NAFL and NASH in comparison to healthy controls. To our knowledge, this is the largest study with biopsy-proven NAFLD patients and healthy controls in the field of gut microbiota.

We observed dysbiosis of the gut microbiota in NAFLD patients. There was no significant difference in the ecological diversities (alpha and beta) among the NAFL, NASH, and healthy control groups, but there was lower total bacterial diversity and richness in the NAFL and NASH groups than in healthy individuals. The results are similar to prior studies from Asia [16,28,29]. The gut microbiota is a complex ecosystem that comprises two predominant bacterial phyla: Bacteroidetes and Firmicutes, which account for approximately 90% of the whole microbiota [30]. At the phylum level, NAFLD patients had higher levels of Bacteroidetes and lower levels of Firmicutes than healthy individuals, although the difference was not significant. This result is similar to those of Wang [28] and Wong et al. [16], who both examined Asian populations. The possible mechanisms might be explained by the strong positive correlations between fecal Bacteroidetes and the content of deoxycholic acid, D-pinitol, choline, raffinose, and stachyose [31,32], which influence the pathogenesis of NASH. In addition, the fecal Bacteroidetes accumulation is associated with an accumulation of branched-chain fatty acids that are produced by amino acid fermentation, which promote insulin resistance and increase the risk of NASH [33]. Thus, fecal Bacteroides might be detrimental for NAFLD. 

Interestingly, our results were significantly different from those of a prior study, which indicated that NASH patients had lower levels of Bacteroidetes than healthy individuals from western countries [15]. Dietary composition is known to influence the growth of the Bacteroides, and Yatsunenko et al. found that a high-fat diet affects the growth [34]. Western pattern diets are generally characterized as being rich in fat, animal proteins, and sugar, while agrarian-society diets are low in red meat and animal-based fats and are rich in whole grains, starch, and plant polysaccharides. This type of diet is traditionally followed by people living in eastern countries such as Taiwan. Hence, we supposed that this discrepancy might partly reflect the different dietary compositions between western and eastern countries. 

In the present study, the ratio of Firmicutes to Bacteroidetes was not different among patients with NAFL or NASH and healthy controls. This result is similar to those of Mouzaki et al. [15], who found that the ratio in NASH patients was not higher than in simple steatosis patients and healthy controls after carefully addressing the potential confounding effects of diet and BMI. Interestingly, in the present study, the ratio was lower in patients with NAFL or NASH than in healthy controls. This is different from the study by Ley et al. [30], in which a reduced proportion of Bacteroidetes and increase in Firmicutes were indicated as main features of obesity-related intestinal dysbiosis in humans. However, this result is still different in the subgroup analysis stratified by BMI (Table S6), in which individuals with BMI ≥25 had significantly higher proportions of Bacteroidetes and less Firmicutes (that is, lower ratios of Firmicutes to Bacteroidetes). Thus, it is still controversial to use the ratio of Firmicutes to Bacteroidetes to evaluate obesity or NASH patients due to the inconsistent results.

One study indicates that the genus *Ruminococcus* is associated with NAFLD, especially in patients with advanced fibrosis [19]. However, we did not find this; instead, we found a lower abundance of the genus *Ruminococcaceae UCG-010* in NAFL and NASH patients than in healthy controls. In the study by Bouraier et al., there was a positive correlation between *Ruminococcus* abundance and significant (F ≥ 2) liver fibrosis [19]. There were 27 patients with advanced fibrosis (F ≥ 2) in that study, but in our cohort, there were only six patients. The different results might be due to the differences in severity between cohorts. Notably, the genus *Ruminococcaceae UCG-010* belongs to the family Ruminococcaceae, order Clostridiales, and class Clostridia, and all of these levels were statistically less abundant in patients with NAFL or NASH than in healthy individuals. Ruminococcaceae can produce high levels of short-chain fatty acids, which can reduce inflammation. Therefore, the lower abundance of Ruminococcaceae may contribute to pathologies associated with NAFL and NASH. To date, no study has examined the genus *Ruminococcaceae UCG-010*. Further research is needed to confirm whether *Ruminococcaceae UCG-010* plays a critical role in the pathogenesis of NAFL and NASH.

We predicted that bacterial functions would be enriched in LPS biosynthesis and fatty acid biosynthesis in NASH patients using PICRUSt analysis. The intestinal microbiota is believed to be the primary source of the bacterial endotoxin LPS produced by gram-negative bacteria. Normally, these bacterial molecules cross the mucosa in only trace amounts, enter the portal blood, and are cleared in the liver. LPS and other endotoxins may influence intestinal permeability, leading to greater translocation of microbial products, such as LPS, into the portal circulation. This would stimulate nuclear factor κB (NF-κB), which recruits inflammatory cells by increasing levels of tumor necrosis factor (TNF) and IL-1β, thus leading to fibrosis in NAFLD [35]. 

In addition to LPS, fatty acid synthase catalyzes the last step in fatty acid biosynthesis. Thus, it is believed to be a major determinant of the maximal hepatic capacity to generate fatty acids by de novo lipogenesis. Dorn et al. indicated that fatty acid synthase may serve as a therapeutic target for the progression of NAFLD [36], suggesting that the pathway of fatty acid biosynthesis plays an important role in the pathogenesis of NAFLD.

Recently, emerging evidence suggests that oral antidiabetic drugs, such as metformin, acarbose, and sitagliptin monotherapy, may respectively alter the composition of gut microbiota and selectively increased the beneficial bacteria [37]. In the present study, there was a total of 12 subjects with diabetes mellitus (two in healthy controls; three in NAFL group; seven in NASH group). Among them, three cases had metformin monotherapy and the other nine cases had combination therapy with metformin, acarbose, and/or sitagliptin. The case number is too small to draw the effects of the antidiabetics on the gut microbiota composition. Thus, whether antidiabetic drugs monotherapy plays a detrimental or beneficial role in the NAFLD patients warrants further investigation.

It is worth noting that our population was well phenotyped by a diagnostic liver biopsy for liver lesions, including the NAFL, NASH, and healthy control groups. NAFLD is easily over- or under-diagnosed by usual clinical and serological parameters. Therefore, studies that have compared individuals with biopsy-proven NASH to a no-biopsy obese control group might have been biased by un-diagnosed NASH among the controls. Currently, there are few studies with biopsy-proven NAFL or NASH [15,16,17,18], and only one study was conducted with biopsy-proven healthy individuals [15]. Therefore, the present study has a homogeneous, well characterized and correctly diagnosed population by biopsy-proven NAFL patients, NASH patients, and healthy individuals in Asia that gives value to the results. 

We explored the relationship between the gut microbiota and the NAFLD using a large cohort and detailed phenotyping of biopsy-proven NAFLD and healthy controls. Nevertheless, additional work is needed to address several limitations. First, the utilization of 16S rRNA gene sequencing rather than metagenomics sequencing is a disadvantage of the study that limits data interpretation in terms of species level and function analysis. Second, our findings could be influenced by confounders such as age, dietary intake, and medication. Although patients using antibiotics or proton pump inhibitors were excluded in our analysis, the confounding effect of medications was probably not sufficiently assessed. Third, all subjects were ethnic Taiwanese, and the findings cannot be directly extrapolated to other populations. However, host regional variation had a strong effect on gut microbiota composition [38]. Finally, this is a cross-sectional study, so it does not show a direct impact of microbiota on the progression of NAFL and NASH. It is relatively difficult to draw conclusions regarding causality with this design. In a future study, it is imperative to use germ-free animal models and additional biofunctional assays to reveal the cause–effect relationship between the significant targeted bacteria and NAFLD.

## 5. Conclusions

In summary, this study has examined the largest number of biopsy-proven NAFL and NASH patients from Asia and the dysbiosis of gut microbiota between NAFLD patients and healthy controls. The diversity pattern was different from those observed in western countries. In addition, we showed the significant depletion of the genus *Ruminococcaceae UCG-010* in the NAFLD patients for the first time. Future studies are required to investigate the biofunction and mechanisms underlying the interaction between the gut microbiota and NAFLD.

## Figures and Tables

**Table 1:** Characteristics of the study population.

	Healthy Controls (Group 1) (*n* = 25)	NAFLD (Group 2 and 3) (*n* = 50)	*p* Value	NAFL (Group 2) (*n* = 25)	NASH (Group 3) (*n* = 25)	*p* Value
Males, n (%)	12 (48)	24 (48)	1.000	13 (52)	11 (44)	0.571
Age (years)	36.7 ± 15.0	51.2 ± 15.0	<0.001	45.8 ± 16.2	56.6 ± 11.6	0.006
BMI (kg/m^2^)	24.8 ± 5.2	31.3 ± 8.9	<0.001	31.2 ± 8.9	31.4 ± 9.0	0.764
Diabetes mellitus, n (%)	2 (8)	10 (20)	0.184	3 (12)	7 (28)	0.162
Hypertension, n (%)	1 (4)	20 (40)	0.001	8 (32)	12 (48)	0.253
AST (U/L)	24.4 ± 14.1	36.0 ± 24.0	0.11	29.0 ± 20.0	43.0 ± 26.0	0.013
ALT (U/L)	22.5 ± 15.7	50.2 ± 43.1	<0.001	36.4 ± 31.4	64.0 ± 49.0	0.005
Total bilirubin (mg/dL)	0.6 ± 0.3	0.8 ± 0.4	0.07	0.7 ± 0.4	0.8 ± 0.4	0.194
Albumin (g/dL)	4.2 ± 0.4	4.6 ± 0.3	<0.001	4.5 ± 0.3	4.6 ± 0.2	0.322
Creatinine (mg/dL)	0.7 ± 0.2	0.9 ± 0.8	0.123	1.0 ± 1.1	0.8 ± 0.2	0.445
Total cholesterol (mg/dL)	176.2 ± 30.1	199.3 ± 40.0	0.009	207.5 ± 44.0	191.8 ± 35.2	0.227
Triglycerides (mg/dL)	97.6 ± 55.0	148.2 ± 61.5	<0.001	131.4 ± 67.5	163.6 ± 52.1	0.021
Fasting glucose (mg/dL)	94.8 ± 15.5	112.0 ± 28.6	0.001	105.6 ± 35.6	118.0 ± 24.8	0.008
Glycohemoglobin (%)	5.6 ± 0.5	6.5 ± 1.4	<0.001	6.1 ± 1.4	6.9 ± 1.2	0.005
Insulin (uU/mL)	9.9 ± 9.2	17.3 ± 23.5	0.002	10.7 ± 5.2	23.2 ± 31.2	0.001
Insulin/GLU	0.08 ± 0.04	0.16 ± 0.24	0.012	0.1 ± 0.04	0.2 ± 0.3	0.004
HOMA-IR	2.0 ± 1.2	4.8 ± 6.1	0.002	2.7 ± 1.6	6.6 ± 8.0	0.002
Steatosis (%)	0 (0–1)	30 (10–46.25)	<0.001	10 (7.5–30)	40 (27.5–55)	<0.001
Fibrosis (F0/F1/F2/F3)	25/0/0/0	28/16/5/1	<0.001	23/2/0/0	5/14/5/1	<0.001
NAS	0	3 (0–4)	<0.001	0 (0–2)	4 (4–5)	<0.001

Data are expressed as mean ± standard deviation, median (interquartile range), or number (percentage). Abbreviations: ALT, alanine aminotransferase; AST, alanine aminotransferase; BMI, body mass index; HOMA-IR, homeostatic model assessment of insulin resistance; NAFLD, nonalcoholic fatty liver disease; NAFL, nonalcoholic fatty liver; NASH, nonalcoholic steatohepatitis; NAS, NAFLD activity score.

## Supplementary Materials

The following are available online at https://www.mdpi.com/2072-6643/12/3/820/s1, Figure S1: The diversity in gut microbiota of healthy individuals and patients with NAFL or NASH. (A) Rarefaction curves of all 16S amplicon samples of all samples. (B) Venn diagram of operational taxonomic units (OTUs) in three groups, Figure S2: Box plots presenting the Firmicutes/Bacteroidetes ratio comparison among patients with nonalcoholic fatty liver disease (NAFL) or nonalcoholic steatohepatitis (NASH) and healthy controls. There was no significant difference among groups. Boxplots shows the median, minimum, and maximum of values, and the Kruskal-Wallis test was performed, Table S1: Relative abundance of bacteria at the phylum level among healthy individuals and patients with nonalcoholic fatty liver disease (NAFL) or nonalcoholic steatohepatitis (NASH), Table S2: Relative abundance of bacteria at the class level among healthy individuals and patients with NAFL or NASH., Table S3: Relative abundance of bacteria at the order level among healthy individuals and patients with NAFL or NASH., Table S4: Relative abundance of bacteria at the family level among healthy individuals and patients with NAFL or NASH, Table S5: Relative abundance of bacteria at the genus level among healthy individuals and patients with NAFL or NASH, Table S6: Relative abundance of bacteria at the phylum level stratified by body mass index (BMI).
