# Supplementary material for: Gut Microbiota Dysbiosis in Patients with Biopsy-Proven Nonalcoholic Fatty Liver Disease: A Cross-Sectional Study in Taiwan
Source: Nutrients. 2020 Mar 19;12(3):820. doi: 10.3390/nu12030820 (PMC7146257; doi:10.3390/nu12030820)
Supplement: Supplementary file 1 [file nutrients-12-00820-s001.pdf]

nutrients

MDPI

Supplementary Materials

Table S1. Relative abundance of bacteria at the phylum level among healthy individuals and patients with NAFL or NASH.

	Healthy Controls (n = 25)	NAFL (n = 25)	NASH (n = 25)	p Value
Bacteroidetes	49.1	52.1	53.7	0.471
Firmicutes	38.7	33.9	33.2	0.17
Proteobacteria	8.0	8.2	9.0	0.577
Fusobacteria	1.3	4.1	2.8	0.091
Verrucomicrobia	1.4	1.1	0.5	0.73
Actinobacteria	1.2	0.6	0.7	0.84
Synergistetes	0.1	0.0	0.0	0.018
Tenericutes	0.1	0.0	0.0	0.353
Lentisphaerae	0.1	0.0	0.0	0.078
Patescibacteria	0.0	0.0	0.0	0.714
Others	0.0	0.0	0.0	0.231

Data are expressed as percentages. Abbreviations: NAFL, nonalcoholic fatty liver; NASH, nonalcoholic steatohepatitis.

Table S2. Relative abundance of bacteria at the class level among healthy individuals and patients with NAFL or NASH.

	Health Controls (n = 25)	NAFL (n = 25)	NASH (n = 25)	p Value
Bacteroidia	49.1	52.1	53.7	0.471
Clostridia	28.5	21.7	18.7	0.005
Negativicutes	9.7	11.7	13.6	0.074
Gammaproteobacteria	7.1	7.8	8.6	0.559
Fusobacteriia	1.3	4.1	2.8	0.091
Verrucomicrobiae	1.4	1.1	0.5	0.073
Actinobacteria	0.8	0.4	0.4	0.307
Deltaproteobacteria	0.7	0.3	0.4	0.056
Bacilli	0.1	0.2	0.7	0.043
Coriobacteriia	0.4	0.2	0.3	0.415
Others	0.8	0.3	0.3	0.059

Data are expressed as percentages. Abbreviations: NAFL, nonalcoholic fatty liver; NASH, nonalcoholic steatohepatitis.

Table S3. Relative abundance of bacteria at the order level among healthy individuals and patients with NAFL or NASH.

	Healthy Controls (n = 25)	NAFL (n = 25)	NASH (n = 25)	p Value
Bacteroidales	49.1	52.1	53.7	0.471
Clostridiales	28.5	21.7	18.7	0.005
Selenomonadales	9.7	11.7	13.6	0.074
Betaproteobacteriales	3.8	4.5	3.5	0.81
Enterobacteriales	3.2	3.1	4.2	0.909
Fusobacteriales	1.3	4.1	2.8	0.091
Verrucomicrobiales	1.4	1.1	0.5	0.089
Bifidobacteriales	0.8	0.4	0.4	0.26
Desulfovibrionales	0.7	0.3	0.4	0.056
Lactobacillales	0.1	0.2	0.6	0.054
Others	1.4	0.7	1.5	0.517

Data are expressed as percentages. Abbreviations: NAFL, nonalcoholic fatty liver; NASH, nonalcoholic steatohepatitis.

Table S4. Relative abundance of bacteria at the family level among healthy individuals and patients with NAFL or NASH.

	Healthy Controls (n = 25)	NAFL (n = 25)	NASH (n = 25)	p Value
Bacteroidaceae	31.1	35.0	29.6	0.420
Prevotellaceae	12.6	12.6	19.1	0.134
Ruminococcaceae	15.2	8.9	9.6	0.019
Lachnospiraceae	12.6	12.2	8.9	0.072
Veillonellaceae	5.6	5.5	7.7	0.153
Acidaminococcaceae	4.1	6.2	6.0	0.148
Burkholderiaceae	3.8	4.5	3.5	0.810
Enterobacteriaceae	3.2	3.1	4.2	0.909
Fusobacteriaceae	1.3	4.1	2.8	0.082
Tannerellaceae	2.0	2.5	2.4	0.946
Others	8.6	5.3	6.2	0.104

Data are expressed as percentages. Abbreviations: NAFL, nonalcoholic fatty liver; NASH, nonalcoholic steatohepatitis.

Table S5. Relative abundance of bacteria at the genus level among healthy individuals and patients with NAFL or NASH.

	Healthy Controls (n = 25)	NAFL (n = 25)	NASH (n = 25)	p Value
Bacteroides	31.1	35.0	29.6	0.420
Prevotella_9	8.6	9.3	11.4	0.236
Faecalibacterium	8.5	5.5	6.2	0.083
Phascolarctobacterium	2.9	4.9	5.0	0.084
Sutterella	3.4	4.1	3.0	0.746
Escherichia-Shigella	2.4	2.5	3.6	0.937
Fusobacterium	1.3	4.1	2.8	0.071
Megamonas	1.9	1.9	4.3	0.006
Lachnoclostridium	2.7	3.4	1.8	0.024
Parabacteroides	2.0	2.5	2.4	0.946
Others	35.4	27.0	30.0	0.114

Data are expressed as percentages. Abbreviations: NAFL, nonalcoholic fatty liver; NASH, nonalcoholic steatohepatitis.

Table S6. Relative abundance of bacteria at the phylum level stratified by BMI.

	BMI ≤ 25 (n = 26)	BMI > 25 (n = 49)	p Value
Bacteroidetes	46.0	54.6	0.001
Firmicutes	39.3	33.1	0.028
Proteobacteria	9.8	7.7	0.028
Fusobacteria	1.6	3.3	0.180
Verrucomicrobia	2.1	0.4	0.208
Actinobacteria	1.1	0.7	0.907
Synergistetes	0.1	0.0	0.349
Tenericutes	0.0	0.0	0.251
Lentisphaerae	0.1	0.0	0.160
Patescibacteria	0.0	0.0	0.973
Others	0.0	0.0	0.889

Data are expressed as percentages. Abbreviations: BMI, body mass index;

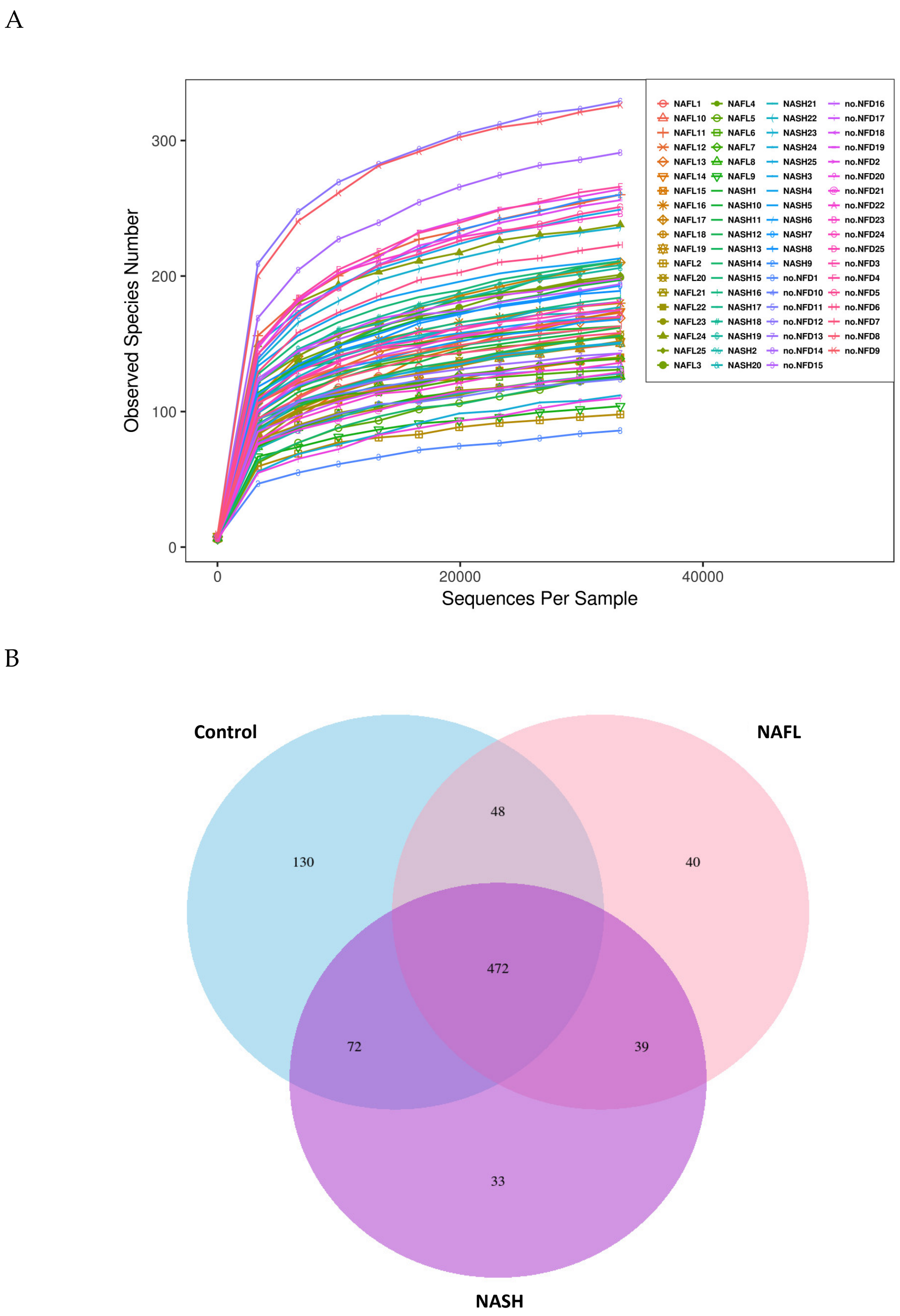

Figure S1. The diversity in gut microbiota of healthy individuals and patients with NAFL or NASH. (A) Rarefaction curves of all 16S amplicon samples of all samples. (B) Venn diagram of OTUs in three groups. NAFL, nonalcoholic fatty liver; NASH, nonalcoholic steatohepatitis.

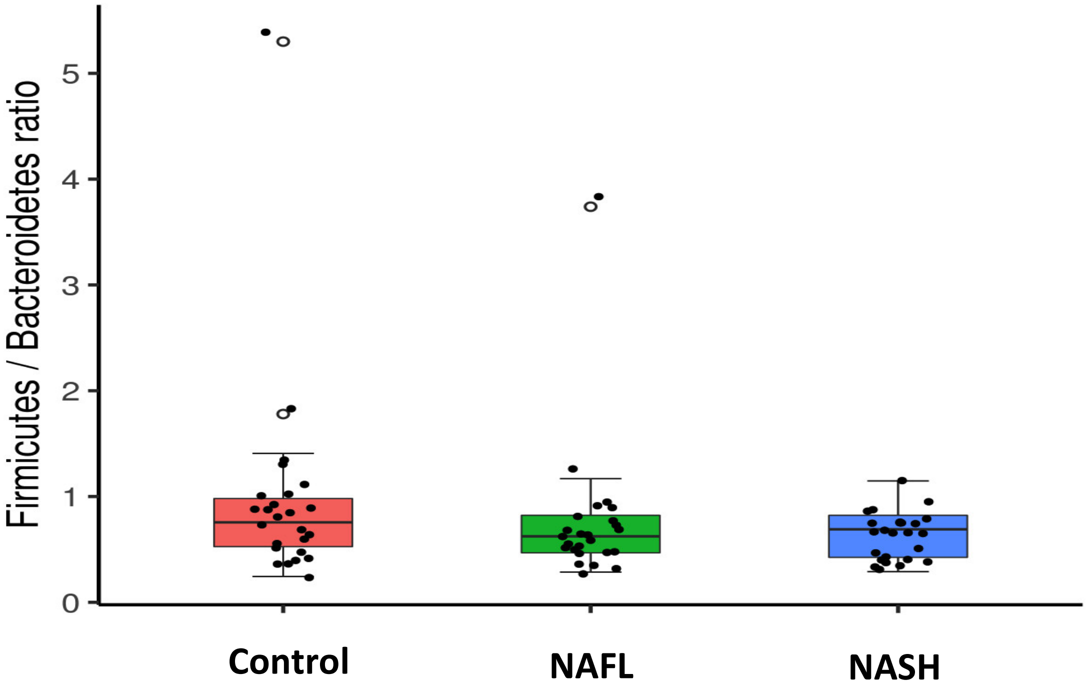

Figure S2. Box plots presenting the Firmicutes/Bacteroidetes ratio comparison among patients with NAFL or NASH and healthy controls. There was no significant difference among groups. Boxplots shows the median, minimum, and maximum of values, and the Kruskal-Wallis test was performed.
